# Optimization of Ultrasound-Assisted Extraction of Flavonoids from *Celastrus hindsii* Leaves Using Response Surface Methodology and Evaluation of Their Antioxidant and Antitumor Activities

**DOI:** 10.1155/2020/3497107

**Published:** 2020-02-15

**Authors:** Dinh-Chuong Pham, Hoang-Chinh Nguyen, Thanh-Hang Le Nguyen, Hoang-Linh Ho, Thien-Kim Trinh, Jirawat Riyaphan, Ching-Feng Weng

**Affiliations:** ^1^Faculty of Applied Sciences, Ton Duc Thang University, Ho Chi Minh City, Vietnam; ^2^Department of Life Science and Institute of Biotechnology, National Dong Hwa University, Shoufeng, Hualien 97401, Taiwan

## Abstract

*Celastrus hindsii* is a potential source of flavonoids with biological activities. This study aimed to develop an ultrasound-assisted technique for extracting flavonoids from leaves of *C. hindsii*. Response surface methodology was employed to optimize the extraction conditions for maximizing the total flavonoid content (TFC). A maximum TFC of 23.6 mg QE/g was obtained under the extraction conditions of ultrasonic power of 130 W, extraction temperature of 40°C, extraction time of 29 min, and ethanol concentration of 65%. The flavonoid-rich extracts were then studied for their antioxidant and anticancer activities. The results showed that the *C. hindsii* leaf extract exhibited potent radical scavenging activities against DPPH (IC_50_ of 164.85 *μ*g/mL) and ABTS (IC_50_ of 89.05 *μ*g/mL). The extract also significantly inhibited the growth of 3 cancer cell lines MCF7, A549, and HeLa with the IC_50_ values of 88.1 *μ*g/mL, 120.4 *μ*g/mL, and 118.4 *μ*g/mL, respectively. Notably, the extract had no cytotoxicity effect on HK2 normal kidney cell line. This study suggests that flavonoid-rich extract is a promising antioxidant and anticancer agent and that ultrasound-assisted extraction is an efficient method for extracting flavonoids from *C. hindsii* leaves.

## 1. Introduction

Cancer is a major health issue, which is ranked as a leading cause of mortality worldwide [1, 2]. To treat cancer, chemotherapy is one of the most commonly used therapeutic methods as it is the most effective approach [3]. However, this approach results in intolerable and serious adverse effects on human health due to the use of synthetic antitumor agents [1]. To address this obstacle, great efforts have been made to develop novel natural products as an alternative with lower toxicity to the host and better effectiveness for the treatment of cancer [4]. In recent years, flavonoids isolated from natural sources have been proven to possess significant health benefits attributed to their antioxidant, anti-inflammatory, and anticancer activities [5–7]. Studies have shown that natural flavonoids are potential agents for the prevention and treatment of cancer [5, 7–9]. Therefore, the search for new and safe flavonoids from natural sources has increasingly attracted considerable attention.

*Celastrus hindsii*, which belongs to the Celastraceae family, is widely distributed in Asia, especially China [10]. *C. hindsii* is commonly used as a traditional medicine to diminish inflammation, cancers, and ulcers [11]. These plants contain various bioactive compounds [10, 11], which exhibits pharmaceutical effects such as antioxidant [11], antiviral [10], and anticancer activities [12, 13]. Considering such medicinal benefits of *C. hindsii*, these plants can be a promising source of flavonoids. However, most previous investigations only focus on the isolation and evaluation of the biological activity of terpenoids, alkaloids, and phenolic compounds [14] whereas studies on the extraction and biological activities of flavonoids from *C. hindsii* are still limited.

Basically, extraction is the first step in the isolation process to obtain bioactive compounds from biomass materials with its purpose to maximize the content of target compounds for their further utilization. To obtain flavonoids from plant materials, several methods have been applied to enhance extraction efficiency [15, 16]. Studies have shown that not only the extraction yield but also the extraction technique is affected by the extracting solvent [17, 18]. The most commonly used methods for flavonoids extraction is boiling, heating, or refluxing [19]. Those methods are time-consuming, have low extraction efficiency, and result in the loss of bioactivities of the flavonoids due to high extraction temperature [19]. To address these problems, microwave-assisted extraction [15] and supercritical fluid extraction [20] have been developed for the extraction of flavonoids [15]. Although those methods efficiently extract flavonoids from plant materials, the microwave-assisted extraction is difficult to scale up and requires high microwave power, which increases energy consumption [15, 21], whereas the supercritical method proceeds at high pressure (48 MPa), which requires expensive reactor and rises a safety concern [20]. Enzyme-assisted extraction has been proposed as another alternative for the flavonoids extraction because this process was performed at mild extraction conditions [22, 23]. However, the high cost of enzyme limits the industrial application of this process. Therefore, it is an urgent need to develop other efficient techniques for extracting flavonoids.

In recent years, the ultrasound-assisted process has gained increased interest in the phytochemical extraction due to its rapid and high extraction efficiency [21, 24]. This method employs ultrasonication, which is ultrasonic waves, to disrupt a cell wall through inducing cavitation in the solvent [21]. This method enhances the mass transfer and solvent penetration into the plant materials, which promote the release of soluble compounds from the plant cell into the extracting solvent [21, 25]. Consequently, this method shortens the extraction time and enhances the extraction efficiency [24]. Remarkably, the ultrasound-assisted extraction process proceeds at low temperatures, thus reducing damage to the molecular and structural properties of extracted compounds [24]. In addition, this process has been proved to be an eco-friendly and economically viable process and easier to scale up for industrial applications as compared to microwave-assisted extraction and supercritical extraction [21, 25]. Ultrasound-assisted extraction has been successfully applied in the extraction of several bioactive compounds such as polysaccharides [21, 24] and total phenolic compounds [15, 17]. However, no study has investigated the use of the ultrasound-assisted technique for the extraction of flavonoids from *C. hindsii*.

Therefore, this work reported the extraction of flavonoids from *C. hindsii* leaves by using an ultrasound-assisted extraction technique. To optimize the extraction conditions, response surface methodology (RSM) was employed to evaluate the effect of extraction factors (ultrasonic power, temperature, extraction time, and ethanol concentration) on the total flavonoids content (TFC). The extract was then studied for its antioxidant and anticancer activities against human breast cancer cell line MCF7, human lung cancer cell line A549, human cervical cancer cell line HeLa, and normal human kidney cell line HK2.

## 2. Materials and Methods

### 2.1. Plant Material

*C. hindsii* (NCBI: txid489979) leaves were collected from the northern area of Vietnam in September 2018 and then identified by the Department of Life Science and Institute of Biotechnology, National Dong Hwa University, Hualien, Taiwan. The leaves were dried in an oven (Memmert, Schwabach, Germany) at 35°C, ground with a stainless-steel grinder (Rong-Tsong, Precision Technology Co., Taiwan), passed through an 80 mesh sieve (W. S. Tyler, Ohio, USA), and kept at 4°C.

### 2.2. Reagents

Reagents were purchased from Merck (Darmstadt, Germany), except when otherwise mentioned. The chemicals including quercetin, Trolox, 2,2-diphenyl-1-picrylhydrazyl (DPPH), and 2,2′-azino-bis(3-ethylbenzthiazoline-6-sulphonic acid) (ABTS) were purchased from Sigma (St. Louis, MO, USA). Dulbecco's Modified Eagle Medium (DMEM), Dulbecco's Modified Eagle Medium/Nutrient Mixture F12 (DMEM/F12), fetal bovine serum (FBS), and antibiotics were obtained from Gibco (Waltham, MA, USA).

### 2.3. Optimization of Ultrasound-Assisted Extraction Using RSM

In this study, total flavonoids were extracted from *C. hindsii* leaves using the ultrasound-assisted extraction technique. Briefly, the *C. hindsii* leaf powder (1 g) was prepared in a 50 mL Erlenmeyer flask containing 20 mL of ethanol and placed into an ultrasonic bath (Branson, Connecticut, USA). To optimize the extraction conditions, a four-factor, three-level Box–Behnken design (BBD) was employed to evaluate the influence of extraction factors on the TFC. Based on the experimental design, ultrasound-assisted extractions were conducted under different ultrasonic power levels (100–200 W), temperatures (30–50°C), extraction times (20–40 min), and ethanol concentrations (50–90%). After the extraction was completed, the resulting solution was centrifuged at 1000 ×g for 10 min and the supernatant achieved was further centrifuged at 15,500 ×g (Beckman Coulter, CA, USA) for 5 min for complete liquid/solid phase separation. The obtained solution was finally evaporated using a rotary evaporator (Heidolph, Germany) and stored at 4°C for further analysis. The relationship between the determined TFC and extraction factors was modeled using equation (1) as follows:

(1) $$\begin{array}{c} Y=\beta_{0}\;+\beta_{1}X_{1}+\beta_{2}X_{2}+\beta_{3}X_{3}+\beta_{4}X_{4}+\beta_{11}X_{1}^{2}+\beta_{22}X_{2}^{2}+\beta_{33}X_{3}^{2}+\beta_{44}X_{4\;}^{2}+\beta_{12}X_{1}X_{2}+\beta_{13}X_{1}X_{3}+\beta_{14}X_{1}X_{4}+\beta_{23}X_{2}X_{3}+\beta_{24}X_{2}X_{4}+\beta_{34}X_{3}X_{4}, \end{array}$$

where *Y* is the TFC (mg QE/g); *X*_1_, *X*_2_, *X*_3_, and *X*_4_ are the ultrasonic power (W), extraction temperature (°C), extraction time (min), and ethanol concentration (%), respectively; *β*_0_ is the intercept coefficient, *β*_1_–*β*_4_ are the linear coefficients, *β*_11_, *β*_22_, *β*_33_, and *β*_44_ are the squared coefficients, and *β*_12_, *β*_13_, *β*_14_, *β*_23_, *β*_24_, and *β*_34_ are the interaction coefficients. Those model coefficients were determined using the least-squares method [26]. Minitab 16 (Minitab Inc., PA, USA) was employed to conduct an analysis of variance (ANOVA), establish the regression model, and determine the optimal extraction conditions for maximizing the TFC.

### 2.4. Determination of TFC

Total flavonoids of the extract were determined by an aluminium chloride colorimetric method [27] with slight modifications. In brief, 500 *μ*L of extract (1 mg/mL) or standard of various concentration solutions was mixed with 1.5 mL methanol, 100 *μ*L of 10% AlCl_3_, 100 *μ*L of 1 M CH_3_COOK, and 2.8 mL distilled water. After 45 min of incubation at room temperature in darkness, the absorbance against blank was obtained at 415 nm using UV-1800 spectrophotometer (Shimadzu, Kyoto, Japan). The TFC was calculated using the calibration curve for quercetin (20–100 *μ*g/mL) as the standard (*y* = 0.009321*x* − 0.003238, *R*^2^ = 0.9929). The result was calculated and expressed in quercetin equivalent per gram of dry weight of crude extract (mg QE/g DW) as follows:

(2) $$\begin{array}{c} F=C\times \frac{V}{M}, \end{array}$$

where *F* is the TFC (QE mg/g of sample), *C* is the concentration of sample established from the calibration curve (mg/mL), *V* is the volume of extract (mL), and *M* is the weight of extract (g).

### 2.5. 2,2-Diphenyl-1-Picrylhydrazyl (DPPH) Assay

The free radical scavenging activity (RSA) of the *C. hindsii* leaf extract was determined using a DPPH test [28] with minor modifications. DPPH was dissolved in 95% ethanol to reach a concentration of 0.2 mM while Trolox, a water-soluble analog of vitamin E, was made at various concentrations of 2–20 *μ*g/mL and used as a positive control. The freshly prepared DPPH stock solution (1 mL) was then added to 1 mL of different concentrations of extract (15.6–1000 *μ*g/mL). The mixtures were shaken and incubated at 37°C for 30 min in darkness, and the absorbance was measured at 517 nm using a UV-1800 spectrophotometer. RSA for DPPH was calculated as

(3) $$\begin{array}{c} \mathrm{inhibition}\;\left(\%\right)=\left(\frac{\left(\text{Ac}-\text{As}\right)}{\text{Ac}}\right)\times 100, \end{array}$$

where Ac is the absorbance of the DPPH without sample and As is the absorbance of the DPPH after adding the sample.

### 2.6. 2,2′-Azino-bis(3-ethylbenzthiazoline-6-sulphonic Acid) (ABTS) Radical Scavenging Assay

The scavenging activity of ABTS^+^ radical cation was measured as reported [29] with some modifications. ABTS^+^ radicals were pregenerated by adding 10 mL of 4.9 mM potassium persulfate to 10 mL of 14 mM ABTS^+^ and kept for 16 h at room temperature in darkness. The working solution was freshly prepared by taking a volume of the previous solution and diluting it in ethanol to yield an absorbance of 0.70 ± 0.023 at 734 nm. To determine the scavenging activity, ABTS^+^ working solution (1.5 mL) was mixed with various concentrations of samples (0.5 mL, 15.6–1000 *μ*g/mL) and the reaction mixture was shaken well and kept for 6 min at room temperature. The absorbance was recorded at 734 nm and the percentage inhibition of the samples was calculated as

(4) $$\begin{array}{c} \text{inhibition}\left(\%\right)=\left(\frac{\left(\text{Ac}-\text{As}\right)}{\text{Ac}}\right)\times 100, \end{array}$$

where As means the absorbance of a sample and Ac means the blank control solution without a sample. Trolox, with a final concentration range of 2–20 *μ*g/mL, was prepared as a positive control.

### 2.7. Cell Culture

Human breast cancer cell line MCF7, human lung cancer cell line A549, human cervical cancer cell line HeLa, and normal human kidney cell line HK2 were purchased from American Type Culture Collection (ATCC, Rockville, MD, USA). MCF7, A549, and HeLa cancer cells were cultured in DMEM supplemented with 10% FBS, 1% antibiotics (100 U/mL of penicillin and 100 *μ*g/mL of streptomycin). HK2 normal cells were grown in DMEM/F12 supplemented with 10% FBS, 1% antibiotics (100 U/mL of penicillin and 100 *μ*g/mL of streptomycin), and 5 ng/mL of human recombinant epidermal growth factor (EGF, Gibco, Waltham, MA, USA). All of the cell lines were kept at 37°C in a humidified atmosphere of 5% CO_2_ incubator.

### 2.8. MTT Assay

Cell viability was analyzed using a colorimetric assay based on 3-(4, 5-dimethylthiazol-2-yl)-2,5-diphenyltetrazolium bromide (MTT), as previously mentioned [30]. Briefly, all of the cell lines were seeded at 8 × 10^3^ cells per well in 96-well plate for 24 h at 37°C in a humidified atmosphere of 5% CO_2_ to allow cell attachment. Cells were treated with a concentration range of 3.1–200 *μ*g/mL of *C. hindsii* leaf extract or 0.31–20 *μ*g/mL of cisplatin for 72 h, and then 20 *μ*L/well MTT (5 mg/mL) solution was added to the wells and further incubated for additional 4 h. The supernatant was decanted, and dimethyl sulfoxide (DMSO, 100 *μ*L/well) was added to allow formazan solubilization. The optical density (OD) value was measured at 570 nm using an Envision microplate reader (Perkin Elmer, Waltham, MA, USA). The percentage of viable cells was determined from a comparison with untreated control. All experiments were performed in triplicate.

### 2.9. Statistical Analysis

The analysis was performed using GraphPad Prism 5 (San Diego, CA, USA). The data obtained were analyzed with Student's *t*-test or one-way analysis of variance (ANOVA). The data are expressed as the mean ± SD. Differences were designated as statistically significant when *p* < 0.05.

## 3. Results and Discussion

### 3.1. RSM Model Development

A Box–Behnken RSM model with four input variables at three levels was applied to investigate the effect of extraction factors (ultrasonic power, extraction temperature, extraction time, and ethanol concentration) on the TFC.  Table 1 shows the coded and uncoded values of the input variables. To obtain the optimal conditions, the extractions were conducted based on the experimental design.  Table 2 presents the experimental results for the extraction. The results showed that the central experiments (run numbers 25–27) exhibit a low coefficient of variance (0.15%), indicating reproducibility and desirable accuracy of the experiments. Consequently, the relationship between input variables and measured responses could be modeled using the pseudoinverse technique and is shown as follows:

(5) $$\begin{array}{c} Y=23.52–0.79X_{1}–0.35X_{2\;}–0.32X_{3}–0.39X_{4}–1.09X_{1}^{2\;}-1.65X_{2}^{2\;}–2.29X_{3}^{2\;}–0.91X_{4}^{2}–0.4X_{1}X_{2\;}–0.01X_{1}X_{3}-0.2X_{1}X_{4}-1.16X_{2}X_{3}-0.25X_{2}X_{4}+0.12X_{3}X_{4}, \end{array}$$

where *X*_3_*X*_4_ had a positive effect on the measured response, while the rest of the parameters have an adverse influence.

**Figure 1:**
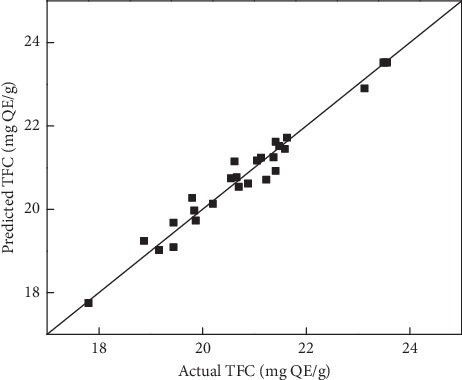
Correlation between predicted and experimental TFC extracted from *C. hindsii* leaves.

Table 3 illustrates the ANOVA for the RSM model. A low *p* value (<0.0001) of the developed model indicated that the model was statistically significant at the 95% confidence level. In addition, the quality of the developed model was examined using the coefficient of determination (*R*^2^). The *R*^2^ value was 0.97, indicating a good correlation between experimental and predicted values, as shown in Figure 1. Furthermore, *t*-tests were used to analyze the significance of each coefficient of the model.

As can be seen from Table 4, small *p* values (<0.05) were obtained for the intercept, all linear coefficients (*X*_1_, *X*_2_, *X*_3_, and *X*_4_), all quadratic coefficients, and an interaction coefficient (*X*_2_*X*_3_), indicating the significance of those corresponding factors in the extraction. Therefore, the developed model is sufficient for plotting response surface curves and predicting optimal extraction conditions for maximizing TFC.

### 3.2. Mutual Effect of Extraction Factors

**Figure 2:**
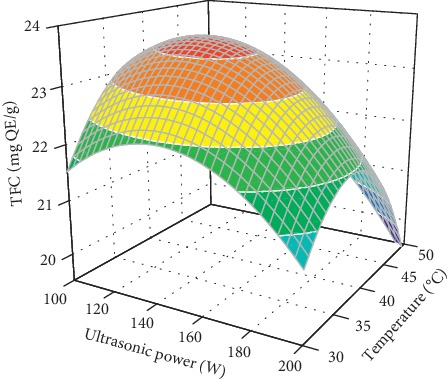
Combined effects of the ultrasonic power and temperature on TFC at an extraction time (30 min) and ethanol concentration (70%).

Figure 2 presents the combined effect of the ultrasonic power and extraction temperature on the TFC while maintaining the extraction time and ethanol concentration at a constant value. There was no interaction between the ultrasonic power and the extraction temperature. At any temperature, the TFC increased when increasing ultrasonic power. This was because a higher ultrasonic power generated a higher cavitation effect, which efficiently broke the plant tissues and cell walls, thus increasing the release of flavonoids into the solvent [16]. However, a further increase in ultrasonic power resulted in a reduction in the TFC. This might be because of the degradation of the extracted flavonoids caused by high ultrasonic power, thus reducing the TFC. This result is in agreement with that for ultrasound-assisted extraction of flavonoids from olive leaves [16] and ultrasound-assisted extraction of polysaccharides from *Artemisia selengensis* [21]. As shown in Figure 2, the ultrasound-assisted extraction is most effective for flavonoids extraction when the ultrasonic power was at its central level.

**Figure 3:**
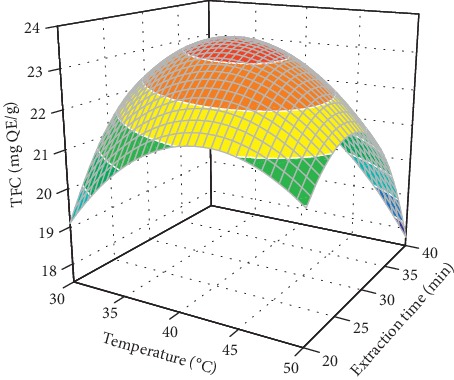
Combined effects of the temperature and extraction time on the TFC at constant ultrasonic power (150 W) and ethanol concentration (70%).

Figure 3 shows the combined effect of temperature and extraction time on the TFC when the remaining factors were maintained at their *constant* level. The TFC is significantly enhanced with the increase in extraction temperature at any extraction time. This was attributed to the positive effect of temperature on the extraction [16, 31]. However, a higher temperature caused a reversal of the aforementioned trend. This result is consistent with the ultrasound-assisted extraction of flavonoids from olive leaves [16] and cocoa shells [31], polysaccharides extraction from *A. selengensis* [21], and the ultrasound-assisted extraction of phenolic from *Trapa quadrispinosa* [15]. This could be because flavonoids are sensitive to temperature and therefore high temperature causes degradation of flavonoids [16, 31]. Similar to temperature, the extraction time also had a significant effect on the TFC. Increasing the extraction time significantly increased the TFC at any extraction temperature. Nevertheless, extended extraction time led to a reduction in the TFC due to the degradation of flavonoids [16, 31], which is similar to that obtained for flavonoids extraction from olive leaves [16] and cocoa shells [31]. In addition, the result is similar to the polysaccharides extraction from *A. selengensis* [21] and phenolic extraction from *T. quadrispinosa* [15].

**Figure 4:**
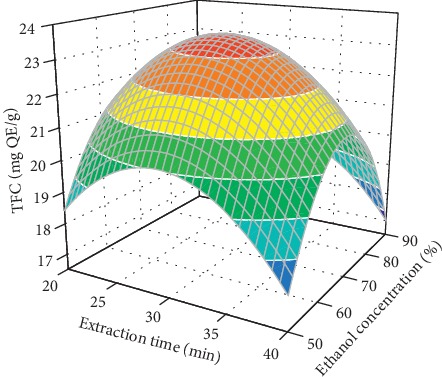
Combined effects of the extraction time and ethanol concentration on the TFC at constant ultrasonic power (150 W) and temperature (40°C).

Figure 4 presents the combined effect of extraction time and ethanol concentration on the TFC while maintaining other factors at a constant level. At a given extraction time, the TFC significantly increased with ethanol concentration. When a maximum TFC was obtained, a further increase in ethanol concentration induced a decrease in the TFC, which is consistent with the result of the flavonoids extraction from cocoa shells [31], *Dendrobium officinale* [32], and *Bombyx batryticatus* [7]. Studies have shown that ethanol concentration is one of the most important factors affecting the extraction of flavonoids [7, 31, 32]. Changing the ethanol concentration adjusts the polarity of the extraction solvent and alters the solubility of flavonoids, thus affecting the efficiency of the flavonoids extraction [32]. In this study, the optimal ethanol concentration for the flavonoids extraction was found to be at its central level.

### 3.3. Obtaining Optimal Extraction Conditions

By solving the RSM model (equation (5)), the maximal TFC was predicted to be 23.74 mg QE/g at an ultrasonic power of 130 W, extraction temperature of 40°C, extraction time of 29 min, and ethanol concentration of 65%. To validate the prediction, experiments were performed under the optimal conditions. The actual TFC was 23.60 ± 0.31 mg QE/g, which was in agreement with the model prediction. This result indicated that the developed RSM model is practicable and can be used to describe the relationship between extraction factors and TFC in the ultrasound-assisted extraction of flavonoids from *C. hindsii* leaves. In addition, the TFC obtained in this study was found to be comparable to that in tomato pulp (21.52 mg QE/g) [33] and much higher than that in *Dysphania ambrosioides* (0.06 mg QE/g) [34] and other medicinal plants [35, 36]. The study suggests that *C. hindsii* leaves are a promising source of flavonoids and the ultrasound-assisted extraction is an efficient process for extracting flavonoids from *C. hindsii* leaves to maximize TFC in a short extraction time and low temperature.

### 3.4. Antioxidant Activities of the *C. hindsii* Leaf Extracts

Oxidative stress is defined as an imbalance between the production of reactive oxygen species (ROS) and a biological system's antioxidant defenses which may involve the development of various diseases including cancer, Parkinson's disease, and heart failure [37]. Antioxidants effectively neutralize free radicals and thus offer the protection from the abovementioned diseases. Regardless of strong activity, synthetic antioxidants may cause harmful side effects and hence the exploration for natural antioxidants has been immensely reinforced in recent years. In the previous studies, plant flavonoids have been shown to own robust antioxidant effects [35, 36]. Because oxidative stress is a noticeably complicated process, different assays were used in examining the antioxidant effect of *C. hindsii* leaf extract.

**Figure 5:**
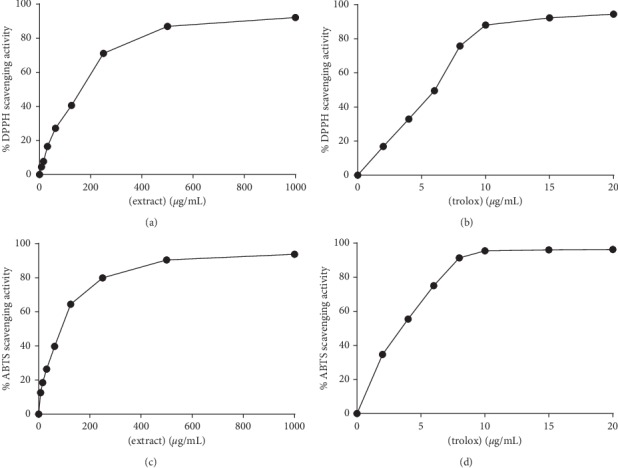
Effect of various concentrations of *C. hindsii* leaf extract (a and c) and Trolox (b and d) in free radical scavenging tests: (a and b) DPPH assay and (c and d) ABTS assay.

The DPPH and ABTS assays are the simple, rapid, and sensitive methods broadly used to evaluate the free radical scavenging activity of numerous plants, pure compounds, and foods. Both DPPH and ABTS are relatively stable free radicals with purple and dark blue-colored when dissolving in ethanol, and their colors were absorbed robustly at 517 nm or 734 nm, respectively. The DPPH and ABTS assay solutions become yellow and lighter blue-colored as an antioxidant scavenges the DPPH and ABTS to the stable forms by hydrogen donation. As shown in Figures 5(a) and 5(b), *C. hindsii* leaf extract and Trolox as the positive control scavenged DPPH radical in a dose-dependent manner by which the DPPH inhibition percentage value increased in the range of the examined concentrations. The IC_50_ values (the concentration of the sample required to inhibit 50% of free radicals) for *C. hindsii* leaf extract and Trolox were 164.85 *μ*g/mL and 5.68 *μ*g/mL, respectively. The DPPH scavenging activity of *C. hindsii* leaf extract in this research was found to be higher than that in *Coriandrum sativum* leaf extract (389 *μ*g/mL) [38] but much lower than that in *Burkea africana* bark extract (7.4 *μ*g/mL) [39]. Figures 5(c) and 5(d) show the scavenging effect of the *C. hindsii* extract and Trolox on the ABTS free radicals increasing with the concentration, and there was a positive equivalence between the ABTS inhibition percentage rate and the concentration of the extract or Trolox. The IC_50_ values of the *C. hindsii* leaf extract and Trolox were 89.05 *μ*g/mL and 3.88 *μ*g/mL, respectively. Dai et al. [40] previously examined ABTS scavenging activity of *Panax notoginseng* stem leaf and reported its IC_50_ value of 855 *μ*g/mL, which was much higher than that in *C. hindsii* leaf extract in the current study. Overall, the results proved that the *C. hindsii* leaf extract is a practicable and efficient DPPH and ABTS free radical scavenger.

### 3.5. Cytotoxic Effect of *C. hindsii* Leaf Extract

In this research, the cytotoxic effects of *C. hindsii* leaf extract on 3 types of cancer cell lines (MCF7, A549, and HeLa) and 1 type of normal cell line (HK2) were studied in 7 serial concentrations ranging from 3.1 *μ*g/mL to 200 *μ*g/mL. The data are shown in Table 5. The results indicated that *C. hindsii* extract significantly (*p* < 0.05) induced the cytotoxicity in MCF7, A549, and HeLa cancer cell lines while exerting no apparent damage to HK2 normal kidney cell line (98% cell viability at 200 *μ*g/mL for 72 h incubation). The IC_50_ values of MCF7, A549, and HeLa cells were 88.1 ± 2.1 *μ*g/mL, 120.4 ± 4.1 *μ*g/mL, and 118.4 ± 2.4 *μ*g/mL at 72 h, respectively. On the other hand, cisplatin, one of the most active antitumor agents, caused the severe cytotoxicity in A549 and HeLa cancer cells or HK2 normal cells with IC_50_ values of 2.3 ± 0.4 *μ*g/mL, 6.7 ± 0.9 *μ*g/mL, and 1.9 ± 0.7 *μ*g/mL, respectively, but much less cytotoxicity in MCF7 cancer cells (70.5% cell viability at 20 *μ*g/mL for 72 h incubation). Such findings demonstrated that *C. hindsii* leaf extract can strongly induce the cytotoxicity of cancer cells but not normal cells.

## 4. Conclusions

This study reports the extraction of flavonoids from leaves of *C. hindsii* by using an ultrasound-assisted technique. Through RSM, the optimal extraction conditions were determined. Under optimal extraction conditions, a maximum TFC of 23.6 mg QE/g was obtained. The antioxidant and anticancer activities of the *C. hindsii* leaf extract were then evaluated. The extract showed a significant inhibitory activity against DPPH (IC_50_ = 164.85 *μ*g/mL), ABTS (IC_50_ = 89.05 *μ*g/mL), and 3 cancer cell lines MCF7 (IC_50_ = 88.1 *μ*g/mL), A549 (IC_50_ = 120.4 *μ*g/mL), and HeLa (IC_50_ = 118.4 *μ*g/mL) but had no cytotoxic effect on the normal kidney cell line HK2. This study suggests that *C. hindsii* leaf extract is a potential antioxidant and anticancer agent for further pharmaceutical use.

## Figures and Tables

**Table 1:** Coded and uncoded values of the variables for RSM optimization.

Variables	Symbols	Variable levels		
		−1	0	1
Ultrasonic power (W)	*X*_1_	100	150	200
Extraction temperature (°C)	*X*_2_	30	40	50
Extraction time (min)	*X*_3_	20	30	40
Ethanol concentration (%)	*X*_4_	50	70	90

**Table 2:** Box–Behnken design matrix in coded values and experimental results.

Run	Variable				Response, *Y*
	*X*_1_	*X*_2_	*X*_3_	*X*_4_	
1	1	–1	0	0	20.55 ± 1.13
2	1	0	–1	0	19.44 ± 1.13
3	1	0	0	–1	21.41 ± 1.01
4	1	0	0	1	20.70 ± 0.87
5	–1	0	0	–1	23.13 ± 1.27
6	0	–1	–1	0	19.44 ± 0.28
7	0	0	1	1	19.87 ± 0.21
8	–1	0	–1	0	21.13 ± 1.01
9	–1	1	0	0	21.41 ± 1.36
10	0	1	1	0	17.80 ± 0.63
11	0	1	–1	0	21.23 ± 0.87
12	0	–1	0	–1	21.59 ± 1.76
13	0	0	–1	1	20.20 ± 0.65
14	0	0	1	–1	19.80 ± 0.38
15	–1	0	0	1	21.63 ± 0.53
16	–1	0	1	0	20.88 ± 0.45
17	1	0	1	0	19.16 ± 0.48
18	0	1	0	1	19.84 ± 0.70
19	1	1	0	0	18.87 ± 1.04
20	–1	–1	0	0	21.48 ± 0.84
21	0	–1	1	0	20.66 ± 0.65
22	0	–1	0	1	21.05 ± 1.16
23	0	0	–1	–1	20.62 ± 0.77
24	0	1	0	–1	21.37 ± 1.34
25	0	0	0	0	23.56 ± 1.49
26	0	0	0	0	23.52 ± 1.41
27	0	0	0	0	23.49 ± 1.56

**Table 3:** Analysis of variance for the RSM model.

Source	DF^b^	SS^b^	MS^b^	*F* value	Probability (*p*) > *F*
Model^a^	14	51.39	3.67	25.87	<0.0001
Residual (error)	12	1.70	0.14		
Total	26	53.09			

^a^Coefficient of determination (*R*^2^) = 0.97; adjusted *R*^2^ = 0.93. ^b^DF, degree of freedom; SS, sum of squares; MS, mean square.

**Table 4:** Significance of the coefficients in the empirical model.

Model term	Parameter estimate	Standard error	*t* value^a^	*p* value
*β*_0_	23.52	0.22	108.17	0.001^b^
*β*_1_	−0.79	0.11	−7.30	0.001^b^
*β*_2_	−0.35	0.11	−3.26	0.007^b^
*β*_3_	−0.32	0.11	−2.98	0.011^b^
*β*_4_	−0.39	0.11	−3.55	0.004^b^
*β*_11_	−1.09	0.16	−6.69	0.001^b^
*β*_22_	−1.65	0.16	−10.14	0.001^b^
*β*_33_	−2.29	0.16	−14.01	0.001^b^
*β*_44_	−0.91	0.16	−5.60	0.001^b^
*β*_12_	−0.40	0.19	−2.14	0.054
*β*_13_	−0.01	0.19	−0.04	0.969
*β*_14_	0.20	0.19	1.05	0.315
*β*_23_	−1.16	0.19	−6.17	0.001^b^
*β*_24_	−0.25	0.19	−1.31	0.213
*β*_34_	0.12	0.19	0.65	0.528

^a^ *t*_*α*/2*,n*-*p*_ = *t*_0.025,12_ = 2.18. ^b^*p* < 0.05 indicates that the model terms are significant.

**Table 5:** Cytotoxic activity of *C. hindsii* leaf extract.

IC_50_ values of antitumor effect of *C. hindsii* leaf extract (*μ*g/mL)				
Sample	MCF7	A549	HeLa	HK2
*C. hindsii*	88.1 ± 2.1	120.4 ± 4.1	118.4 ± 2.4	>200
Cisplatin	>20	2.3 ± 0.4	6.7 ± 0.9	1.9 ± 0.7

The results are expressed as mean ± SD of three independent experiments (*n* = 3).

## Data Availability

The data used to support the findings of this study are included within the article.
